# Duplicating a tandem and ovoids distribution with intensity‐modulated radiotherapy: a feasibility study

**DOI:** 10.1120/jacmp.v8i3.2450

**Published:** 2007-07-17

**Authors:** Harish K. Malhotra, Jaiteerth S. Avadhani, Steven F. de Boer, Wainwright Jaggernauth, Michael R. Kuettel, Matthew B. Podgorsak

**Affiliations:** ^1^ Department of Radiation Medicine Roswell Park Cancer Institute Buffalo New York U.S.A.

**Keywords:** HDR brachytherapy, IMRT, DMLC

## Abstract

Brachytherapy plays an important role in the definitive treatment of cervical cancers by radiotherapy. In the present study, we investigated whether sliding‐window intensity‐modulated radiation therapy (IMRT) can achieve a pear‐shaped distribution with a similar sharp dose falloff identical to that of brachytherapy. The computed tomography scans of a tandem and ovoid patient were pushed to both a high dose rate (HDR) and an IMRT treatment planning system (TPS) after the rectum, bladder, and left and right femoral heads had been outlined, ensuring identical structures in both planning systems. A conventional plan (7 Gy in 5 fractions, defined as the average dose to the left and right point A) was generated for HDR treatment. The 150%, 125%, 100%, 75%, 50%, and 25% isodose curves were drawn on each slice and then transferred to the IMRT TPS. The 100% isodose envelope from the HDR plan was the target for IMRT planning. A 7‐field IMRT plan using 6‐MV X‐ray beams was generated and compared with the HDR plan using isodose conformity to the target and 125% volume, dose– volume histograms, and integral dose. The resulting isodose distribution demonstrated good agreement between the HDR and IMRT plans in the 100% and 125% isodose range. The dose falloff in the HDR plan was much steeper than that in the IMRT plan, but it also had a substantially higher maximum dose. Integral dose for the target, rectum, and bladder were found to be 6.69 J, 1.07 J, and 1.02 J in the HDR plan; the respective values for IMRT were 3.47 J, 1.79 J, and 1.34 J. Our preliminary results indicate that the HDR dose distribution can be replicated using a standard sliding‐window IMRT dose delivery technique for points lying closer to the three‐dimensional isodose envelope surrounding point A. Differences in radiobiology and patient positioning between the two techniques merit further consideration.

PACS: 87.53.Jw

## I. INTRODUCTION

Brachytherapy plays an important role in the definitive treatment of cervical cancers. A pear‐shaped dose distribution with an extremely sharp dose gradient is usually achieved using a tandem and ovoid applicator with the appropriate arrangement of sources (or dwell positions). The original low dose rate brachytherapy application involved a dose rate of approximately 60 cGy/h to point A, and this application is the baseline against which all other distributions are compared. However, that original brachytherapy procedure had many shortcomings, including lengthy treatment time and hospital admission to deliver a tumoricidal dose. Further drawbacks include applicator movement errors, patient discomfort, use of limited radioactive source patterns, and inability to use patient‐specific source configurations (inventory, source leakage, and administrative requirements).

The advent of high dose rate (HDR) afterloading technology reduced the treatment time, enabling outpatient treatment. Moreover, HDR provides better immobilization and displacement of dose‐sensitive organs for the short treatment times. However, the transition from low dose rate to HDR brachytherapy has not been uniformly accepted because of radiobiologic differences and a lack of consensus on the equivalent number of fractions and dose per fraction. Moreover, a HDR afterloading system has its own problems, including high capital cost and special shielded room design, among other considerations. Furthermore, in developed countries, the incidence of cancer of cervix is comparatively low, and because of early detection, the rate of presentation of advanced‐stage lesions requiring radiation and, therefore, brachytherapy as treatment modality is even lower. These factors have combined to produce a scenario in which not all treatment centers can provide the brachytherapy option to patients. Recently, delivery of this type of therapy with an intensity‐modulated radiation therapy (IMRT) approach has been proposed.^(^ ^1^ ^–^ ^5^ ^)^

Since the late 1990s, the number of centers offering IMRT to their patients has grown tremendously. The IMRT treatment planning process involves contouring the tumor and the associated structures in the form of gross tumor volume, clinical target volume (CTV), planning target volume, and so on—including organs at risk (OARs). Using either a predefined or customized beam arrangement and the required dose constraints for tumor and OARs, an optimal plan is generated by the inverse treatment planning system. The deliverable plan is then generated from the optimal plan by making corrections for the physical limitations of the available multileaf collimator (MLC) on the treatment unit. The MLC leaf width, leaf speed [for dynamic MLC (DMLC) IMRT], penumbra, and interleaf and intraleaf MLC transmission are some of the parameters that limit the maximum dose gradient with an IMRT dose delivery setup. Accordingly, concerns arose about whether the standard high dose gradient associated with brachytherapy could be achieved with the IMRT dose delivery technique. In the present study, we determined the feasibility of a sliding‐windows IMRT dose delivery technique (DMLC) to give an equivalent intracavitary isodose distribution characterized by a sharp dose gradient.

## II. MATERIALS AND METHODS

After the rectum, bladder, left and right femur heads, and other structures had been contoured, the computed tomography (CT) scans of a patient with an implanted tandem and ovoid applicator were electronically transferred to a Plato treatment planning system (version 14.2: Nucletron, Veenendaal, Netherlands) for HDR planning and to an Eclipse treatment planning system (version 7.3.10: Varian Medical Systems, Palo Alto, CA) for IMRT planning. This approach ensured that identical structure sets were used in both planning systems.

The applicator (central tandem and two ovoids) was reconstructed using CT‐reconstruction options available on the Plato system. A conventional brachytherapy treatment plan (7 Gy in 5 fractions) was generated for the HDR with dose prescribed at the average of left and right point A doses. A total of 13 active dwell positions were used in the HDR plan, including 3 in each ovoid. A 5‐mm step size was used. The 150%, 125%, 100%, 75%, 50%, and 25% isodose curves were computed on each slice and then transferred to the Eclipse TPS as additional structure sets. As a result, the HDR isodose curves could instantly be visualized against the equivalent IMRT plan.

For IMRT planning purposes, the isodose curve representing the 100% isodose line from the HDR treatment plan was defined as the target. For structures representing isodose values obtained from the HDR treatment plan, the dose constraints were designed in such a way that 100% of the volume (for example, ${\text{CTV}}_{100}/\text{target}$) would receive the minimum dose (100% of the dose). For all the isodose structures, the maximum dose constraint was set to 150% of the prescribed dose. This constraint was necessary because of the overlapping nature of the structures based on the successive isodose curves.

**Figure 1:**
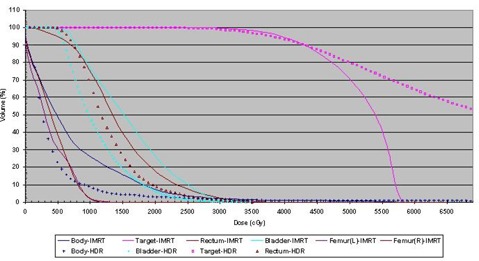
Dose‐volume histograms for some of the structures for both the high dose rate (HDR) plan and the 6‐MV 7‐field intensity‐modulated radiation therapy plan.

The IMRT plan was designed to give an equivalent prescription dose of 35 Gy in 5 fractions. The necessary constraints for rectum and bladder were determined from the HDR dose‐volume histogram [DVH (Fig. 1)]. An IMRT plan with 7 coplanar fields (gantry angles 0, 30, 100, 130, 220, 260, and 330 degrees) using 6‐MV X‐rays was used for optimization. The linear accelerator had a 52‐leaf MLC with a leaf width of 1 cm each at the isocenter. The IMRT plan employed the sliding windows technique. Comparison of the two isodose distributions (HDR and IMRT) was carried out in the three orthogonal planes. An independent comparison of the plans based on the DVHs, dose conformity index, and integral dose^(6)^ was also carried out. In the present study, “conformity index” was defined as the ratio of the target volume covered by the reference isodose to the target volume defined as in report 62 of the International Commission on Radiation Units and Measurements.^(7)^ Mathematically,

$$\text{conformity}\;\text{index}=V_{\text{RI}}/TV\;\;,$$

where $V_{\text{RI}}$ represents the volume of the reference isodose, and *TV* stands for the target volume.

## III. RESULTS

**Figure 2:**
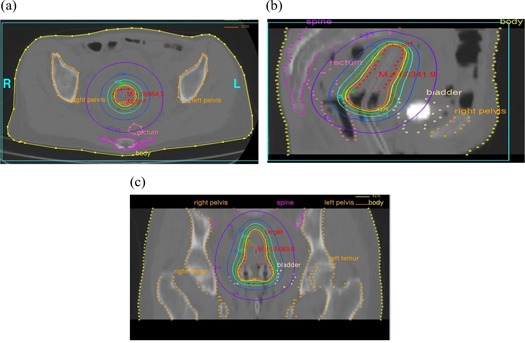
Isodose distribution generated using the standard high dose rate brachytherapy plan in (a) transverse section, (b) sagittal section, and (c) coronal section. The plotted isodose lines are 5250 cGy, 4375 cGy, 3500 cGy, 2625 cGy, 1750 cGy, and 875 cGy, which correspond to 150%, 125%, 100%, 75%, 50%, and 25% of the prescription dose.

**Figure 3:**
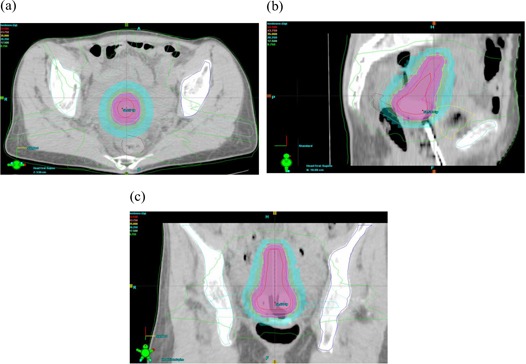
Isodose distribution generated using a 6‐MV 7‐field intensity‐modulated radiation therapy (IMRT) plan in (a) transverse section, (b) sagittal section, and (c) coronal section. The isodose curves from the IMRT plan are shown as solid lines; the respective high dose rate distributions are shown as color washes.

Fig. 2(a–c) shows the isodose distribution generated using the standard HDR brachytherapy plan in the transverse, sagittal, and coronal planes. The plotted isodose lines represent 5250 cGy, 4375 cGy, 3500 cGy, 2625 cGy, 1750 cGy, and 875 cGy, which correspond to 150%, 125%, 100%, 75%, 50%, and 25% of the prescription dose. The transverse distribution is at the point A level, and the sagittal cut is at the central tandem level. Fig. 3(a–c) shows the respective orthogonal distributions obtained using IMRT dose delivery technique. In that figure, the 150%, 125%, 100%, 75%, 50%, and 25% isodose curves from the IMRT isodoses have been shown as solid lines, and the respective HDR isodoses have been shown as color washes.

**Figure 4:**
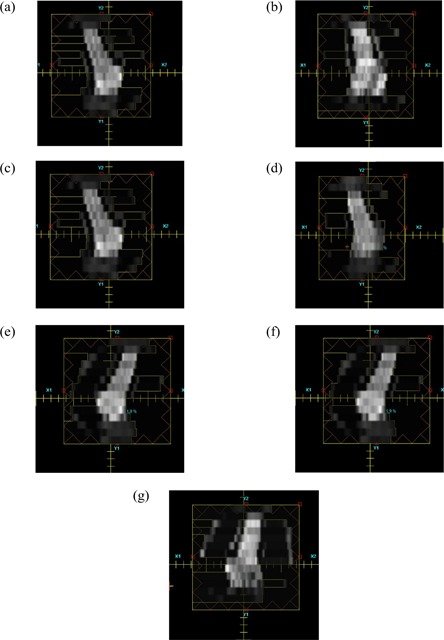
Fluence map for the various intensity‐modulated radiation therapy fields. (a) Field 1 (gantry 0 degrees). (b) Field 2 (gantry 30 degrees). (c) Field 3 (gantry 100 degrees). (d) Field 4 (gantry 130 degrees). (e) Field 5 (gantry 220 degrees). (f) Field 6 (gantry 260 degrees). (g) Field 6 (gantry 330 degrees).

Fig. 1 shows the DVHs for some of the structures for HDR, and the 6‐MV 7‐field IMRT plan. The dose falloff in the HDR plan is much steeper, but it also has a substantially higher maximum dose. Integral doses for the target, the rectum, and the bladder are found to be 6.69 J, 1.07 J, and 1.02 J, respectively for the HDR plan, and 3.39 J, 1.62 J, and 1.13 J for the IMRT plan. The conformity index of the IMRT plan for the target (100% isodose envelope from the HDR plan) is 0.97. Figure 4(a–g) shows the respective deliverable fluence maps for the 7 fields used in IMRT dose delivery.

## IV. DISCUSSION

Analysis of Fig. 3 shows nice conformality of the IMRT isodose distributions with the HDR isodose distributions (shown as color washes). The isodose curves in the HDR and IMRT plans were also evaluated slice by slice, and for isodose values higher than or equal to 75%, excellent agreement between the plans was observed. Below the 75% isodose level, the agreement was not as good, probably because of the limited number of beams used in the plan. The match was acceptable within the active field area, but lower in the region between fields, which received only scatter and leakage dose. This good agreement between the two plans satisfies the desired clinical range within the vicinity of point A, where the difference between the IMRT 100% isodose envelope and the HDR 100% isodose line was just 1 mm or less.

The isodose distribution around a brachytherapy source in three dimensions is governed by the shape of the radioactive source after accounting for its anisotropy. For a point source, this distribution is spherical in nature. The conventional limited‐field teletherapy plan may provide an identical shape because of cross‐firing at high isodose values, but in the area in between two successive fields that are matched at tumor, there will be a region (maximum at skin) that is receiving only scatter and leakage radiation. This component is much less in magnitude in the region between fields, as compared with the radiation level along the central axis, because of the predominant forward scattering with the megavoltage beams being used. In contrast, with the use of brachytherapy sources, all areas around each source receive primary radiation in addition to scatter. Thus, at low isodose values, brachytherapy still provides a good amount of dose in the region between adjacent therapy fields, but teletherapy does not. As the number of beams increases, more and more beams add to the primary component of the radiation in the patient. In a rotation therapy, where the gap at skin between adjacent fields disappears, all parts of the patient receive primary, scatter, and leakage radiation, yielding an isodose pattern that may produce better conformity of the IMRT isodose with the HDR isodose envelope even at lower isodose levels.

Comparison of integral doses for the HDR and IMRT plans for the target, the rectum, and the bladder produce interesting results. The integral dose values were found to be 6.69 J, 1.07 J, and 1.02 J, respectively for the HDR plan, and 3.39 J, 1.62 J, and 1.13 J for the IMRT plan. The difference in integral dose could be attributable to the very nature of the brachytherapy, which necessitates a very high dose in the immediate vicinity of the radioactive source (essentially infinity at zero distance). Whether such high doses are clinically necessary or are a necessary evil of brachytherapy is not clear. These high values very near to the source are never even recorded in a clinical brachytherapy practice. In contrast, in teletherapy, every effort is made during the planning stage to reduce hotspots. We therefore feel that the congruence at prescription isodose (100%±25%] may provide a better index for comparison between the plans generated from two different modalities.

The conformity index of the IMRT plan for the target (100% isodose envelope from the HDR plan) was 0.97, which indicates very good conformity between the two plans. It is important to note that the conformity index is a scalar quantity (ratio of volumes) and that, by itself, it cannot be the true measure of conformity.^(8)^ Accordingly, we analyzed the isodose distributions for the two plans slice by slice on the same distribution (isodose values for the HDR plan as color washes, and isodose values for the IMRT plan as solid lines) and found excellent congruence.

It is important to note that the present study is retrospective in nature. Therefore, even though the overlay comparison of the IMRT isodose lines and the HDR isodose envelopes provides better perspective, the method is difficult to use prospectively because of the absence of the HDR isodose envelopes during the IMRT planning stage. Nevertheless, brachytherapy tandem‐and‐ovoid distributions are geometric in nature: they depend on the tandem length and ovoid diameter. Thus, determining expected volumes for the isodose curves for future patients is not difficult once an atlas of these plans as a function of tandem length and ovoid diameter is in place. We are currently working on solving these issues, and a follow‐up paper will dwell on them in more detail.

The actual delivery of a brachytherapy plan has many potential sources of error, including steep dose gradients. The absence of CT‐ or magnetic resonance–compatible applicators in most centers necessitates treatment planning based on orthogonal X‐rays, which do not provide the volumetric organ information (tumor and OARs) that are otherwise standard in IMRT plans. Even in centers that do have applicators, additional time and effort are needed to contour the various organs and subsequently generate a treatment plan (although this additional time and effort in contouring the structures is common to any IMRT‐based treatment). In HDR, identifying the first dwell position in every catheter—preferably with an accuracy better than ±1 mm—is very important. Achieving this accuracy in a clinical setup is difficult, because in most centers, CT scans are taken at 3 – 5 mm intervals. Furthermore, not all brachytherapy planning systems account for the presence of shields within the ovoids, thereby putting a question mark on the accuracy of reported dose values for various critical structures.

A possibility also exists of significant differences between actual dose delivery and the treatment plan because of applicator movement attributable to unavoidable patient motion during the various stages of brachytherapy (simulation, treatment, and so on). The biggest shortcoming of IMRT‐based treatment plans is the localization accuracy of cervix and uterus during daily fractions. With the growing clinical acceptance of image‐guided IMRT‐capable cone‐beam CT‐based LINACs and tomotherapy machines, localization accuracy may cease to be an issue in the very near future. Better immobilization, localization, three‐dimensional dose computation algorithms incorporating heterogeneity corrections, and daily treatment verification will make it technically possible to treat such patients with a noninvasive IMRT dose delivery technique that provides brachytherapy‐equivalent distribution without the associated time constraints inherent in brachytherapy.

## V. CONCLUSIONS

Our preliminary results indicate that HDR distribution can be replicated using standard IMRT for all points lying close to point A, although the DVHs of rectum and bladder are not identical (a slightly higher dose is shown for those structures). Nevertheless, smaller facilities that lack HDR brachytherapy on site could offer equivalent IMRT treatments instead. The radiobiologic and patient positioning differences between the two techniques merit further consideration. However, the latter can be easily handled using standard pre‐treatment imaging options available with treatment units having image‐guided radiotherapy capabilities.

## Supporting information

Supplementary Material

Supplementary Material
