# Supplementary material for: Duplicating a tandem and ovoids distribution with intensity‐modulated radiotherapy: a feasibility study
Source: J Appl Clin Med Phys. 2007 Jul 17;8(3):91–8. doi: 10.1120/jacmp.v8i3.2450 (PMC5722607; doi:10.1120/jacmp.v8i3.2450)
Supplement: Supplementary file 1 — Supplementary Material [file ACM2-8-091-s001.doc]

# Duplicating a Tandem and Ovoids Distribution with IMRT: A Feasibility Study

**Harish K. Malhotra, Ph.D., Jaiteerth S. Avadhani, Ph.D., Steven deBoer MS, Wainwright Jaggernauth, MD, Michael Kuettel, MD and Matthew B. Podgorsak, Ph.D.**

*Department of Radiation Medicine*

*Roswell Park Cancer Institute*

*Buffalo, NY, 14263*

[*Harish.Malhotra@Roswellpark.org*](mailto:Harish.Malhotra@Roswellpark.org)

*Suggested running title: Tandem and ovoids with IMRT*
