# Supplementary material for: Duplicating a tandem and ovoids distribution with intensity‐modulated radiotherapy: a feasibility study
Source: J Appl Clin Med Phys. 2007 Jul 17;8(3):91–8. doi: 10.1120/jacmp.v8i3.2450 (PMC5722607; doi:10.1120/jacmp.v8i3.2450)
Supplement: Supplementary file 2 — Supplementary Material [file ACM2-8-091-s002.doc]

# Duplicating a Tandem and Ovoids Distribution with IMRT: A Feasibility Study

**Harish K. Malhotra, Ph.D., Jaiteerth S. Avadhani, Ph.D., Steven deBoer MS, Wainwright Jaggernauth, MD, Michael Kuettel, MD and Matthew B. Podgorsak, Ph.D.**

*Department of Radiation Medicine*

*Roswell Park Cancer Institute*

*Buffalo, NY, 14263*

[*Harish.Malhotra@Roswellpark.org*](mailto:Harish.Malhotra@Roswellpark.org)

*Suggested running title: Tandem and ovoids with IMRT*

**Abstract:**

Brachytherapy plays an important role in the definitive treatment of cervical cancers by radiotherapy. In the present study, we have investigated whether we can achieve identical pear shaped distribution with similar sharp dose falloff using a sliding-window IMRT. The CT scans of a tandem and ovoid patient were pushed to HDR as well as IMRT treatment planning system after drawing the rectum, bladder, left and right femoral heads. This ensured identical structures in both planning systems. A conventional plan (7Gy in 5 fractions defined as the average dose to the left and right point A) was generated for the HDR. The 150, 125, 100, 75, 50 and 25% isodose curves were drawn on each slice and then transferred to IMRT TPS. The 100% isodose envelope from the HDR plan was referred as target for IMRT planning. A 7-field IMRT plan using 6 MV x-ray beams was generated and compared with the HDR plan using isodose conformity to the target & 125% volume, DVHs and integral dose. The resultant isodose distribution demonstrated good agreement between the HDR and IMRT plans in the 100 & 125% isodose range. Though the dose fall off in the HDR plan was much steeper, it also had the maximum dose substantially higher. Integral dose for the target, rectum and bladder were found to be 6.69, 1.07 and 1.02 J for HDR plan while the respective values for IMRT were 3.47, 1.79 and 1.34. Our preliminary results indicate that it is possible to replicate the HDR dose distribution using a standard sliding window IMRT dose delivery technique for points lying closer to 3D isodose envelope surrounding point A. Radiobiological and patient positioning differences between both the techniques merit further consideration.

Keywords: HDR brachytherapy, IMRT, DMLC

**I. Introduction:**

Brachytherapy plays an important role in the definitive treatment of cervical cancers. A pear shaped dose distribution with an extremely sharp dose gradient is usually achieved using a tandem and ovoid applicator with the appropriate arrangement of sources (or dwell positions). The original low-dose-rate brachytherapy application involved a dose-rate of around 60 cGy/hr to point A and is the basis for which all other distributions are compared. This brachytherapy procedure, however, had many shortcomings including lengthy treatment time and hospital admission to give tumorcidal dose. Further drawbacks include applicator movement errors, patient discomfort, use of limited radioactive source patterns, and inability to use patient specific source configurations (inventory, source leakage and administrative requirements). The advent of high-dose-rate (HDR) afterloading technology has reduced the treatment time enabling out-patient treatment and provides better immobilization and displacement of dose sensitive organs for the short treatment times. However, the transition from low-dose rate to high-dose-rate brachytherapy has not been uniformly accepted because of the radiobiological differences and the fact that there is no consensus on the equivalent number of fractions and dose-per fraction. Moreover, an HDR afterloading system has its own problems including high capital cost, special shielded room design etc. Furthermore, in developed countries, the incidence of cancer of cervix is comparatively low and due to its early detection, the lesions which present in the advanced stage requiring radiation and, therefore, brachytherapy as a modality of treatment will be even lower. All these factors have resulted in a scenario that not all treatment centers provide the brachytherapy option to their patients. Recently, it has been proposed to deliver this type of therapy with an IMRT approach (1,2, 3, 4, 5).

In the past decade, there has been tremendous growth in the number of centers offering IMRT to their patients. The IMRT treatment planning process involves contouring of tumor and the associated structures in the form of GTV, CTV, PTV etc including the organs at risk (OARs). Using either a predefined or customized beam-arrangement and the required dose-constraint for tumor and OARs, an optimal plan is generated by the inverse treatment planning system. The deliverable plan is then generated from the optimal plan by making corrections for the physical limitations of the available MLC on the treatment unit. MLC leaf width, leaf speed (for DMLC IMRT), penumbra, inter & intra-leaf MLC transmission are some of the parameters which limit the maximum dose-gradient with an IMRT dose-delivery setup. Accordingly, there were concerns whether we can achieve the standard high dose gradient associated with brachytherapy with IMRT dose delivery technique. In the present study, we have determined the feasibility of sliding windows IMRT dose delivery technique [DMLC] to give an equivalent intracavitary isodose distribution characterized by a sharp dose-gradient.

**II. Materials & Methods:**

After contouring the rectum, bladder and left & right femur heads etc. the CT scans of a patient with an implanted tandem and ovoid applicator were electronically transferred to a Plato treatment planning system (ver. 14.2) for HDR planning and to Eclipse treatment planning system (ver. 7.3.10) for IMRT planning. This ensured identical structure sets in both planning systems. The applicator (central tandem & two ovoids) was reconstructed using CT-reconstruction options available on the Plato planning system. A conventional brachytherapy treatment plan (7 Gy in 5 fractions) was generated for the HDR with dose prescribed at the average of left and right point A doses. A total of 13 active dwell positions were used in the HDR plan including 3 in each ovoid. A 5 mm step-size was used. The 150, 125, 100, 75, 50 and 25% isodose curves were computed on each slice and then transferred to Eclipse treatment planning system as additional structure sets. This allowed instant visualization of HDR isodose curves against equivalent IMRT plan. For IMRT planning purposes, the isodose curve representing the 100% isodose line from the HDR treatment plan was defined as the target. For structures representing isodose values obtained from HDR treatment plan, the dose constraints were designed in such a way that 100% of the volume [e.g. CTV100 / target] gets the minimum dose of 100% of the dose. The maximum dose constraint was set to 150% of the prescribe dose for all the isodose structures. This was necessary due to the overlapping nature of the successive isodose curves based structures. The IMRT plan was designed to give equivalent prescription dose of 35 Gy in 5 fractions. The necessary constraints for rectum and bladder were determined from the HDR DVH [Fig. 3]. A 7 coplanar field IMRT plan (Gantry angles = 0°, 30°, 100°, 130°, 220°, 260° & 330°) using 6 MV x-ray was used for optimization. The linear accelerator had a 52-leaf MLC having a leaf width of 1 cm each at the isocenter. The IMRT plan employed sliding-window technique. Comparison of the two isodose distributions (HDR & IMRT) was carried out in all 3 orthogonal planes. An independent comparison based on the dose-volume histograms, dose-conformity index and integral dose [6] between both the plans was also carried out. In this study, conformity index was defined as the ratio of the target volume covered by the reference isodose and the target volume as defined in Report 62 of the International Commission on Radiation Units and Measurements [7]. Mathematically,

Conformity Index = V_RI_ / TV

Where V_RI_ represents the volume of the reference isodose while TV stands for the target volume.

**III. Results:**

Figure 1(a), 1(b) & 1(c) show the isodose distribution generated using the standard HDR brachytherapy plan in transverse, sagittal and coronal planes, respectively. The plotted isodose lines are of 5250, 4375, 3500, 2625, 1750 & 875 cGy which correspond to 150, 125, 100, 75, 50 and 25% of the prescription dose. The transverse distribution is at the point A level while the sagittal cut is at the central tandem level, respectively. Figure 2(a), 2(b) and 2(c) show the respective orthogonal distributions obtained using IMRT dose delivery technique. In Fig. 2(a), 2(b) and 2 (c), the 150, 125, 100, 75, 50 and 25% isodose curves from the IMRT isodoses have been shown as a solid curves while the respective HDR isodoses have been shown as a color wash.

Figure 3 shows the dose-volume histograms for some of the structures for HDR as well as the 6 MV 7/field IMRT plan. Though the dose fall off in the HDR plan was much steeper, it also had the maximum dose substantially higher. Integral dose for the HDR and IMRT plan for the target, rectum and bladder were found to be 6.69, 1.07 and 1.02 J for HDR while the respective values for IMRT were 3.39, 1.62 and 1.13 J. The conformity index of the IMRT plan for the target [100% isodose envelope from HDR plan] was found to be 0.97. Figure 4 (a-g) shows the respective deliverable fluence map for the 7 fields used in IMRT dose delivery.

**IV. Discussion:**

Analysis of Fig. 2 shows a nice conformality of the IMRT isodose distribution with the HDR isodose distributions which have been shown as a color wash. The isodose curves in the HDR and IMRT plans were also evaluated slice by slice and for isodose values higher than or equal to 75%, there was an excellent agreement between both of them. Below 75% isodose level, the agreement was not as good probably due to the limited number of beams used in the plan. The match was acceptable within the active field area but lower in the region in between fields which gets dose only from scatter and leakage dose. Such a good agreement between both the plans satisfies the desired clinical range within the vicinity of the point A where a difference between the IMRT 100% isodose envelope and the HDR 100% isodose line was up to 1 mm only.

The isodose distribution around a brachytherapy source in 3D is governed by the shape of the radioactive source after accounting for its anisotropy. For a point source such a distribution is spherical in nature. The conventional limited field teletherapy plan may provide identical shape due to cross-firing at high isodose values. In the area in between 2 successive fields which are matched at tumor, there will be region [maximum at skin] which will be getting dose only from scatter and leakage radiation. This component is much less in magnitude in the region in-between fields in comparison with the radiation level along the CAX with present megavoltage beams due to the predominant forward scattering. This is in contrast with brachytherapy sources where all parts around the source get primary radiation regardless besides the scatter. Thus, in between the region of adjacent therapy fields at low isodose values, brachytherapy will still give good amount of dose but not the teletherapy. As the number of beams increase, more and more beams add to the primary component of the radiation in the patient and in a rotation therapy where the gap at skin between the adjacent fields disappears, all parts of the patient get radiation from primary, scatter as well as leakage giving an isodose pattern which may extend better conformity of IMRT isodose levels with HDR isodose envelope even at lower isodose levels.

Comparison of integral dose for the HDR and IMRT plans for the target, rectum and bladder show interesting results. The integral dose values for target, rectum and bladder were found to be 6.69, 1.07 and 1.02 J for HDR plan while the respective values for IMRT were 3.39, 1.62 and 1.13 J. The difference in integral dose could be due to the very nature of the brachytherapy which necessitates a very high dose in the immediate vicinity of the radioactive source [essentially infinity at zero distance]. Whether such high doses are clinically necessary or are a necessary evil of brachytherapy is not clear. These high values very near to the source are never even recorded in a clinical brachytherapy practice which is in contrast to teletherapy where every effort during the planning stage is done to reduce hot-spots. We, therefore, feel that the congruence at prescription isodose [100% ± 25%] may provide a better index for comparison between the plans generated from two different modalities.

The conformity index of the IMRT plan for the target [100% isodose envelope from HDR plan] was found to be 0.97 which shows very good conformity between both the plans. It is important to note that conformity index is a scalar quantity [ratio of volumes] and by itself can not be the true measure of conformity [8]. Accordingly, the isodose distributions between both the plans were analyzed slice by slice on the same distribution [HDR plan isodose values as color wash while IMRT plan isodose values as solid isodose lines] and an excellent congruence between both was noted.

It is important to note that our present study is retrospective in nature. So even though the comparison of IMRT plan isodose lines and HDR isodose envelopes on the same plan provides better perspective, it is difficult to use this method prospectively due to the absence of HDR isodose envelopes during IMRT planning stage. However, it is important to note that brachytherapy tandem and ovoid distributions are geometrical in nature depending on the tandem length and ovoid diameter. Thus, it is not difficult to determine the expected volumes for the isodose curves for future patients once we have an atlas of these plans as a function of tandem length and ovoid diameter. We are presently working on solving these issues and a follow-up paper on the subject will dwell more in detail on it.

There are many potential sources of error in the actual dose-delivery of a brachytherapy plan including steep dose-gradients. The absence of CT/MR compatible applicators in the majority of the centers necessitates treatment plans based on orthogonal X-rays which do not provide the volumetric organ (tumor as well as OAR) information otherwise standard in IMRT plans. Even in the centers which do have these applicators, additional time and effort are needed to contour various organs and subsequently generate a treatment plan. Incidentally this additional time and effort in contouring the structures is common with any IMRT based treatments. In HDR, it is very important to identify the position of the first dwell position in every catheter preferably with accuracy better than ± 1 mm. It is difficult to achieve the same in a clinical set up as in majority of the centers, CT scans are taken at 3-5 mm intervals. Also not all brachytherapy planning systems account for the presence of shields within the ovoids, thereby putting a question mark on the accuracy of reported dose values for various critical structures. There is also a possibility of significant differences in the dose delivery from the treatment plan due to the applicator movement due to the unavoidable patient motion during various stages of brachytherapy (simulation, treatment etc.). The biggest shortcoming of the IMRT based treatment plans is the localization accuracy of cervix & uterus during daily fractions. With the growing clinical acceptance of IGRT IMRT capable cone-beam CT based linacs as well as Tomotherapy machines, this may not be issue in very near future. Because of better immobilization, localization, 3D dose computation algorithms incorporating heterogeneity corrections and daily treatment verification, it will be technically possible to treat such patients with a non-invasive IMRT dose delivery technique with brachytherapy equivalent distribution without the associated time-constraint inherent in brachytherapy.

**V. Conclusion:**

Our preliminary results indicate that it is possible to replicate the HDR distribution using standard IMRT for all points lying close to point A. The DVH of rectum and bladder, though, are not identical and show a little higher dose for these structures. This will allow smaller facilities which do not have HDR brachytherapy on-site to offer equivalent IMRT treatments instead. Radiobiological and patient positioning differences between both the techniques merit further consideration. The latter, however, can be easily handled using standard pre-treatment imaging options available with IGRT capable treatment units.

**References:**

1. Daniel A. Low, Perry W. Grigsby, James F. Dempsey, Sasa Mutic, Jeffrey F. Williamson, Jerry Markman, K.S. Clifford Chao, Eric E. Klein and James A. Purdy. Applicator-guided intensity-modulated radiation therapy. Int. Jr. of Radiat. Oncol. Biol. Phys. 52, 1400-1406 (2004).
2. Schefter TE, Kavanagh BD, Wu Q, Tong S, Newman F, Mccourt S, Arnfield M, Benedict S, and Mohan R. Technical considerations in the application of intensity-modulated radiotherapy as a concomitant integrated boost for locally-advanced cervix cancer. Med. Dosimetry, 27(2): 177-184, 2002.
3. Mundt AJ, Roeske JC, Lujan AE. Intensity-modulated radiation therapy in gynecologic malignancies. Med. Dosimetry, 27(2): 131-136, 2002.
4. Lujan AE., Mundt AJ., Yamada SD, Rotmensch J, Roeske JC. Intensity-modulated radiotherapy as a means of reducing dose to bone marrow in gynecologic patients receiving whole pelvic radiotherapy. Int. J. Radiation Oncology Biol. Phys., 57 (2), 516–521 (2003).
5. Mundt AJ., Lujan AE., Rotmensch J, Waggoner SE., Yamada SD, Fleming G, Roeske JC. Intensity-modulated whole pelvic radiotherapy in women with gynecologic malignancies. Int. J. Radiation Oncology Biol. Phys., 57 (2), 516–521 (2003).
6. Johns, HE, Cunningham, JR. The Physics of Radiology. Fourth Edition. Charles C Thomas. Publisher. 1983.
7. Anonymous. Prescribing, recording and reporting photon beam therapy (supplement to ICRU Report 50). Report 62, International Commission on Radiation Units and Measurements, Washington, DC 1999.
8. Feuvret L, Noel G, Mazeron JJ, Bey P. Conformity index: a review. Int. J. Radiation Oncology Biol. Phys., 64 (2), 333–342 (2006).

**Figure Captions**

Fig. 1: Isodose distribution generated using the standard HDR brachytherapy plan in (a) transverse section, (b) sagittal section and (c) coronal section. The plotted isodose lines are of 5250, 4375, 3500, 2625, 1750 & 875 cGy which correspond to 150, 125, 100, 75, 50 and 25% of the prescription dose.

Fig. 2: Isodose distribution generated using 6 MV 7/field IMRT plan in (a) transverse section, (b) sagittal section and (c) coronal section. The isodose curves from the IMRT plan has been shown as a solid curves while the respective HDR distributions have been shown as a color wash.

Fig. 3: Dose-volume histograms for some of the structures for HDR as well as the 6 MV 7/field IMRT plan.

Fig. 4: Fluence Map for the various IMRT fields. (a) Field 1 (G=0°), (b) Field 2 (G=30°), (c) Field 3 (G=100°), (d) Field 4 (G=130°), (e) Field 5 (G=220°), (f) Field 6 (G=260°), (g) Field 6 (G=330°).
